# Lignin: A Biopolymer from Forestry Biomass for Biocomposites and 3D Printing

**DOI:** 10.3390/ma12183006

**Published:** 2019-09-16

**Authors:** Mihaela Tanase-Opedal, Eduardo Espinosa, Alejandro Rodríguez, Gary Chinga-Carrasco

**Affiliations:** 1 RISE PFI, Høgskoleringen 6b, 7491 Trondheim, Norway; mihaela.tanase@rise-pfi.no; 2 Chemical Engineering Department, Faculty of Science, Universidad de Córdoba, Building Marie-Curie, Campus de Rabanales, 14014 Córdoba, Spain

**Keywords:** lignin, polylactic acid (PLA), 3D printing, biocomposites, biopolymers

## Abstract

Biopolymers from forestry biomass are promising for the sustainable development of new biobased materials. As such, lignin and fiber-based biocomposites are plausible renewable alternatives to petrochemical-based products. In this study, we have obtained lignin from Spruce biomass through a soda pulping process. The lignin was used for manufacturing biocomposite filaments containing 20% and 40% lignin and using polylactic acid (PLA) as matrix material. Dogbones for mechanical testing were 3D printed by fused deposition modelling. The lignin and the corresponding biocomposites were characterized in detail, including thermo-gravimetric analysis (TGA), differential scanning calorimetry (DSC), Fourier transform infrared (FTIR) spectroscopy, X-ray diffraction analysis (XRD), antioxidant capacity, mechanical properties, and scanning electron microscopy (SEM). Although lignin led to a reduction of the tensile strength and modulus, the reduction could be counteracted to some extent by adjusting the 3D printing temperature. The results showed that lignin acted as a nucleating agent and thus led to further crystallization of PLA. The radical scavenging activity of the biocomposites increased to roughly 50% antioxidant potential/cm^2^, for the biocomposite containing 40 wt % lignin. The results demonstrate the potential of lignin as a component in biocomposite materials, which we show are adequate for 3D printing operations.

## 1. Introduction

Environmental pollution and the increasing awareness of limited resources have been a major driver in finding renewable alternatives to replace traditional fossil-based plastics, with bio-based materials (for ex., biocomposites) derived from carbon-neutral feedstocks [1]. Natural fibres have various advantages, including good mechanical properties, no emission of toxic substances, and reduction in cost [2]. Additionally, natural fiber-reinforced biocomposites have attracted increasing attention due to several beneficial properties, e.g., low-cost, good mechanical properties, and lightweight.

Poly (lactic) acid (PLA) is a promising biopolymer which has been introduced commercially as a renewable alternative to fossil-based polymers [3,4]. A number of promising PLA-based products are presently found commercially, such as automotive parts [5]. PLA provides good mechanical properties and relatively easy melt-processability. However, PLA is relatively expensive and some properties (e.g., brittleness) limit the utilization of PLA in some applications [6]. Therefore, additional polymers can be used in PLA blends in order to tailor the properties of the final products [7]. Moreover, some alternatives have been suggested to improve e.g. the mechanical and thermal properties, including the addition of fibre or filler materials [8] and cellulose nanofibres [9].

Lignin as the second most abundant renewable bio-resource, next to cellulose, is considered as a waste product in several industrial processes. Attempts for lignin valorisation have been published in a vast number of papers and reviews over the last years [10,11,12]. 

The notable properties of lignin, such as highly abundance, low-cost and biodegradability, high carbon content, high aromaticity, and reinforcing capability make it a good candidate as a potential component for biocomposites [13]. Each year, over 50 million tons of lignin are produced worldwide as a side product from biorefineries, of which 98% are burned to generate energy. Only 2% of the lignin has been used for other purposes, mainly in applications such as dispersants, adhesives, and fillers [14,15,16,17]. The commercial lignin is mainly lignosulfonates from sulphite pulp mills (about 1 mill. tons/year) and less than 100,000 tons/year of kraft lignin [18]. Since lignin has lower energy content than coal and because lignin-rich side streams are wet, the energy value is limited to 50 US Dollar/dry ton [19]. Thus, cost-efficient valorisation of lignin into value-added products offers a significant opportunity to enhance operational efficiency and generates additional revenue so that the production of bioethanol or other products from the hydrolysed carbohydrates becomes more competitive. However, lignin properties (for ex., high heterogeneity and complex structure), make it difficult to predict how the lignin loading will affect the properties of a given biocomposite. Recent reviews summarized the research done on lignin-reinforced thermoplastic biocomposites [20,21].

Lignin can be used as a component in biocomposites, with or without modification, depending on the target application. Gordobil et al. [22] acetylated kraft lignin to improve the affinity with PLA. However, incorporation of kraft lignin decreased the tensile strength properties of the PLA with increasing lignin loading of 10 wt % and 20 wt %. Furthermore, when acetylated lignin was blended with a thermoplastic the tensile strength was improved [21,22]. Without modification, lignin can be directly incorporated into a polymeric matrix, such as UV-light stabilizer, antioxidant, flame retardant, plasticizer, and flow enhancer to reduce production cost, reduce plastic, and potentially improve material properties [23,24,25,26]. 

Lignin can also be used as a coupling agent in natural fibre biocomposites. Lignin can act as a compatibilizer between the hydrophilic fibres and hydrophobic matrix polymer, thus strengthening the fibre matrix interface [27,28,29]. Lignin treatments of hemp fibres [28] and flax fibres [27] have been shown to improve compatibility between fibres and the thermoset matrix, thus also improving the mechanical properties of the biocomposites. Graupner [29] reported increased tensile properties of compression-moulded PLA-cotton composites when the fibres were treated with lignin.

In our study, lignin was extracted by a soda process. Soda pulping pre-treatment is similar to that of alkaline pulping process, which uses alkali (e.g., NaOH, Ca(OH)_2_) to solubilize or depolymerize lignin, and make lignin extractable from biomass matrix [30]. Soda pre-treatment disrupts the lignin structure and breaks the aryl-ether, ester, and C-C linkages among the lignin and hemicelluloses and hence open up the lignocellulose structure [31]. This process has mainly been studied on herbaceous biomass and to some extent hardwood [12,32,33]. The main difference of this process compared to the chemical pulping process is the moderate treatment severity, separating lignin with low condensation structure [19]. The soda process for obtaining lignin has some essential advantages from the environmental point of view in comparison with the sulphate process, namely, the soda cooking liquor has a lower content of low molecular products of wood degradation, which get to waste water, and the formation of the unpleasant sulphur organic compound odorants is prevented [34]. Soda lignin has found application in multiple areas: the production of phenolic resins [35,36], animal nutrition [37], dispersants [38], and synthesis of polymers [39]. A major difference between soda lignin compared with kraft lignin and lignosulphonates is that soda lignin is sulphur-free, without odour and with a chemical composition closed to pure lignin [38,39]. Additionally, soda lignin derived from non-wood plants contains a larger fraction of carboxyl groups and p-hydroxyl units [39].

Three-dimensional (3D) printing has evolved rapidly in recent years. This technology allows the production of unique, complex and customized structures by digitized and computer assisted processes, reducing production time and costs [40]. In addition, less waste in production and lower chemical consumption is required by this technique in comparison with the traditional processing manufacturing. Fused deposition modelling (FDM) is one of the most used 3D printing technologies, which consists in the melting of thermoplastic materials at high temperature, which are solidified when cooling. Currently, there is great interest in the use of biomass and biomass components for use in 3D printing by FDM. However, unlike petroleum-derived thermoplastic compounds, lignocellulosic components are difficult to melt for the extrusion and injection moulding processes. Therefore, the development of new materials from biomass suitable for 3D printing is a challenge [41,42,43]. The production of pure lignin composites is limited by its high thermal transition temperature and high flow resistance [40]. For this reason, lignin is mixed with other polymers that favour its melting behaviour and flow. Recently, organosolv hardwood lignin [40] and kraft softwood lignin [44] have been applied to manufacture filaments for FDM, based on acrylonitrile-butadiene-styrene and PLA polymers, respectively. 

The purpose of this study was to demonstrate the suitability of PLA/soda lignin biocomposite filament for 3D printing. A motivation for selecting soda lignin is that it is sulphur-free. Soda lignin was thus expected to reduce the typical smell that is experienced when melt-processing biocompounds containing kraft lignin or lignosulfonates. Thus, samples with varying PLA/soda lignin weight ratios were manufactured and the mechanical (tensile testing), thermal (TGA, DSC analysis), morphological (SEM), FTIR, X-ray diffraction, and antioxidant properties were assessed.

## 2. Materials and Methods 

### 2.1. Raw Materials

The lignin used in this study is a softwood lignin extracted from cooking liquor, using a soda cooking process of Norway spruce chips, collected from Norske Skog, Skogn, Norway. The PLA used in this study was a commercial grade for 3D printing (Ingeo PLA 3D850, NatureWorks LCC, Minnetonka, MN, USA).

### 2.2. Lignin Extraction

Lignin was extracted from Norway spruce by soda process using a MK circulation reactor. In this method, 400 g dried chips were cooked with cooking liquor which comprised 30% NaOH (30 g NaOH/100 g dried chips). The liquid wood ratio was 7.5:1. The temperature was increased slowly in order to get a good impregnation of the chips with the cooking liquor. Once the reactor reached the operating temperature of 180 °C, it was maintained for 100 min. After cooling, the cooking liquor was removed from the bottom of the reactor. 

### 2.3. Lignin Precipitation

Sulphuric acid was slowly added to the cooking liquor under agitation until final pH of 2.5. A color change from black to brown was observed at pH 5.5. In addition, a viscosity change was observed, the liquid was more viscose at lower pH. These changes occur due to the initial stage of the lignin precipitation. The mixture was then centrifuged (3500 rpm, 10 min) to recover the lignin. The lignin was repeatedly washed with water, then dried in an oven at 105 °C overnight. Volatile organic compounds (VOC) were removed after drying in a 2L Parr reactor (N4622, Parr Instruments, Illinois, USA).

### 2.4. Filaments and 3D Printing

Neat PLA (100%) and blends of PLA and lignin (20 wt % and 40 wt %) were extruded through a Noztek Xcalibur (Table 1). The temperatures in the three heating chambers were 200 °C, 205 °C, and 210 °C. The speed of the screw extruder was set to 15 mm/s. The target diameter of the filaments was 1.75 mm. The filaments were extruded twice in order to improve the mixing of the PLA and lignin. 

An original Prusa i3 MK3S was used for 3D printing by fused deposition modeling (FDM), using a 0.4 mm nozzle and a printing speed of 35 mm/s. Dogbone samples (length 63 mm, width 3 mm) were printed for mechanical testing. Three sets of dogbones were printed with varying printing temperatures of 205, 215, and 230 °C. The printing direction was 45°. In addition, a smartphone protective case was printed as a demonstration.

### 2.5. Characterisation

The lignin and carbohydrates analyses were performed according to the standard procedures of NREL (NREL/TP–510–42623). The lignin samples were hydrolysed in two steps using sulfuric acid: first step hydrolysis was done with 72 wt % H_2_SO_4_, 30 °C for 60 min, followed by the second step with 4 wt % H_2_SO_4_ at 121 °C for 1 h). The resulting supernatant was filtered (0.2uL) and the filtrates were analysed by High Performance Anion Exchange Chromatography (HPAEC, ICS-5000, Dionex, CarboPac PA1 4 × 250 mm column), for carbohydrates. Klasson lignin (acid insoluble lignin) contents were determined gravimetrically, while acid soluble lignin was determined by using TAPPI method (TAPPI UM 250). The absorbance was measured with UV-VIS spectrometry at 205 nm, ɛ of 110. Carbohydrates were detected on a pulsed amperometry detector (PAD). All carbohydrate contents are reported as anhydrosugars. Purity of lignin samples was calculated from the sum of the ash and sugar results. 

#### 2.5.1. Thermo-Gravimetric Analysis (TGA) and Differential Scanning Calorimetry (DSC) 

TGA was used to determine the thermal stability, decomposition temperature, and char yield for soda lignin, PLA, and different blends of lignin/PLA. The analyses were performed using a Netzsch Jupiter F3 equipment, operating in nitrogen environment. Samples for TGA for each measurement were maintained at 14 ± 5 mg and scans were preformed from 30 °C to 800 °C with the heating rate of 10 °C/min to observe thermal degradation and stability of lignin, PLA, and the corresponding biocomposites. DSC was performed to measure the glass transition temperature (Tg) of soda lignin.

#### 2.5.2. Scanning Electron Microscopy (SEM)

The fracture surface of the dogbones after mechanical testing was sputtered with a layer of gold to make it conductive under the electron beam. Scanning electron microscopy (SEM, Hitachi SU3500, Hitachi High-Technologies Co., Tokyo, Japan) was performed in secondary electron imaging mode using an acceleration voltage of 5 kV and a working distance of 5–6 mm.

#### 2.5.3. Mechanical Testing

Test specimens obtained from the 3D printing process were used for tensile testing of the biocomposites.

The 3D printed dogbones were mechanically tested with a Zwick Roell Proline (Zwick GmbH & Co. KG, Ulm, Germany) and a load cell of 2.5 kN. Four specimens of each series were tested. The speed and the grip distance were 20 mm/min and 50 mm, respectively.

#### 2.5.4. Fourier Transform Infrared (FTIR) Spectroscopy 

FTIR spectra were collected using a spectrometer FTIR-ATR Perkin Elmer Spectrum (Perkin Elmer, Waltham, United States). Two single spectra were collected in the wavelength range from 4000 to 450 cm^−1^ with a resolution of 4 cm^−1^ and a total of 40 scans.

#### 2.5.5. X-ray Diffraction Analysis (XRD)

X-ray diffraction analysis of the test specimens was carried out in a Bruker D8 Discover Instrument (Bruker Corporation, Karlsruhe, Germany) with a monochromatic source (CuKα1) over an angular range of 5−50° at a scan speed of 1.56°/min. 

#### 2.5.6. Antioxidant Activity

The 2,2′-Azino-Bis-3-Ethylbenzothiazoline-6-Sulfonic Acid (ABTS) assay was used to measure the antioxidant activity of the biocomposites. A radical solution (7 mM ABTS and 2.45 mM potassium persulphate) was prepared and left in dark during 14–16 h before testing. The radical solution was adjusted to an absorbance of 0.70 ± 0.02 at 734 nm diluting with ethanol. A specimen of 0.5–1 cm^2^ of the biocomposites was added to a 4 mL of radical solution and the absorbance of the solution was measured at 734 nm using ethanol as blank. The absorbance was measured after 6 min. The antioxidant activity was determined according to the following equation [45]:

(1) $$AOP\;\left(\%\right)=\;\frac{A_{734,ABTS6^{\prime }}-A_{734,sample6^{\prime }}}{A_{734,ABTS0^{\prime }}}\times 100$$

where A_734,ABTS6’_ is the absorbance at 734 nm of the radical solution after 6 min, A_734,sample6’_ the absorbance at 734 nm of the sample after 6 min, and A_734,ABTS0’_ the absorbance at 734 nm of the radical solution before the 6 min. Because the radical scavenging activity was performed in the surface of the biocomposites, the antioxidant activity was related to the surface exposed to the radical solution, expressed as antioxidant potential, (AOP%/cm^2^).

## 3. Results and Discussion

### 3.1. Lignin Composition

The yield of lignin extraction was estimated to be 25%, based on Klasson lignin. The chemical composition shows that lignin is composed of 82% acid insoluble lignin, 6% acid soluble lignin, 1.6% carbohydrates, and ash. Surprisingly, glucomannan, the predominant constituent of softwood hemicellulose, was not found in the soda lignin. However, xylan was found in relatively high amounts. This shows that cleavage of the lignin−carbohydrates complexes is less complete for the soda process applied in this study. These carbohydrates may originate from lignin−carbohydrates complexes or from carbohydrates that are trapped during the lignin precipitation step which end up non-covalently bonds in the lignin after drying [46]. Furthermore, the high ash content in soda lignin may partly originate from the salts resulted after neutralization of cooking liquor during precipitation. These results are in accordance with literature results [47]. The chemical composition of lignin and ash content depends on the feedstock as well as on the isolation process. In general, alkaline lignin contains more residual sugar than other type of lignins [47]. 

The moisture content of isolated lignin was less than 2.5%. Sameni et al. [48] reported that lignin with higher impurities, containing hydrophilic compounds, led to more moisture content. 

### 3.2. TGA

**Figure 1:**
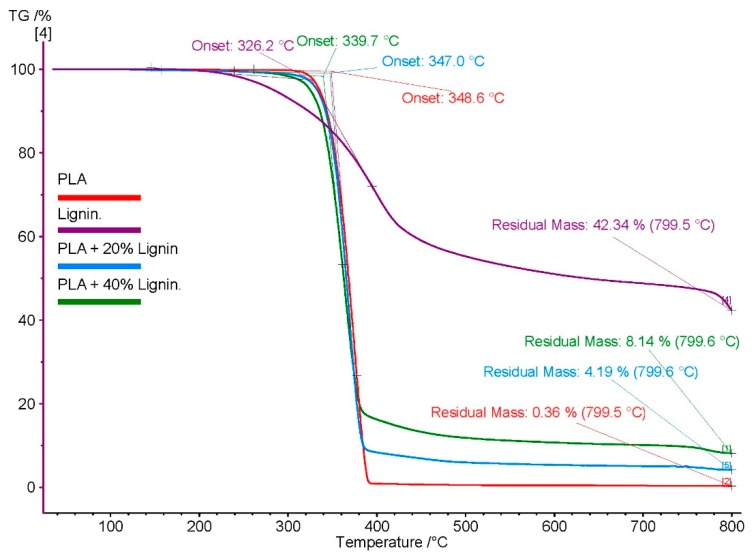
Thermo-gravimetric analysis (TGA) plot of Lignin, polylactic acid (PLA), and PLA/Lignin biocomposites.

TGA curves reveal the residual mass of materials with respect to the temperature of thermal degradation and was used to assess the thermal stability of the both polymers PLA and Lignin but also of the biocomposites, PLA/20%Lignin and PLA/40%Lignin. The different chemical bonds present in the lignin molecular structure, leads to a range of degradation temperatures, extending from 100 to 800 °C [49]. The results show that 42 wt % of lignin sample still remained at 800 °C. This is due to the formation of highly condensed aromatic structures which have the ability to form char (Figure 1). Degradation of lignin sample can be divided into three stages [36]. Firstly, an initial weight loss between 50–120 °C, due to water evaporation. Secondly, above 220 °C degradation of carbohydrates occurs, which are converted to gasses such as CO, CO_2_, and CH_4_ [50]. The last stage occurs to around 420 °C, and then continues to lose mass at a slower rate, leading to the formation of gaseous products and phenolics, alcohols and aldehyde acids [50]. PLA, PLA/20%Lignin, and PLA/40%Lignin present a similar thermal degradation behavior. They show a faster thermal degradation between 340–400 °C. However, the residual mass at 800 °C is higher for biocomposites with lignin due to the carbonaceous composition of the lignin. With respect to the starting temperature of decomposition, the lignin shows a faster degradation (326 °C) compared to neat PLA (348 °C). As expected, the biocomposites resulting from the mixtures PLA/Lignin present an intermediate degradation start temperature (347 °C and 339 °C for 20% and 40% lignin content, respectively).

### 3.3. DSC

**Figure 2:**
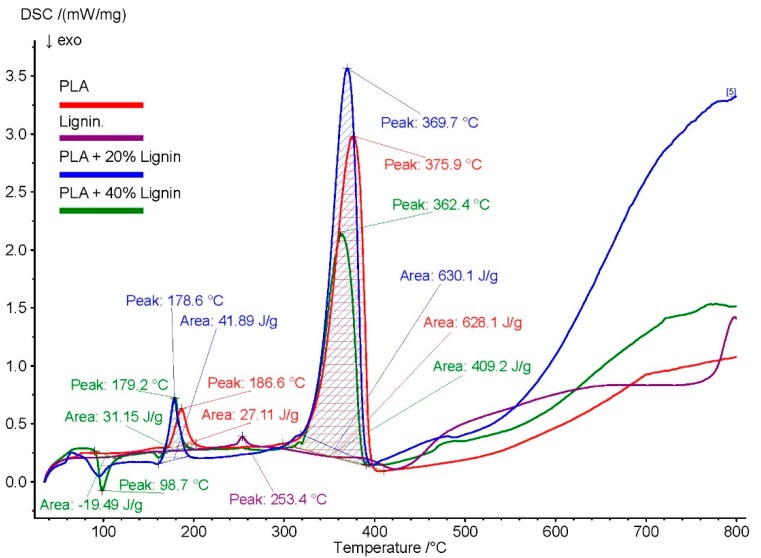
Differential scanning calorimetry (DSC) thermographs of Lignin, PLA, and PLA/lignin biocomposites.

The reaction energy was measured on soda lignin, polymer and blends of lignin and polymer to assess the thermal degradation. Enthalpy measurements were similar for PLA/40%Lignin (27 J/g) compared with neat PLA (31 J/g). At higher a temperature of 376 °C (PLA) the enthalpy of 628 J/g was similar with PLA/20%Lignin and higher than PLA/40%Lignin (362 °C, 409 J/g), see Figure 2. No significant variations in Tm (temperature maximum degradation) were found with the addition of lignin to PLA matrix. The temperature of maximum degradation occurred between 360–400 °C for all biocomposite samples. Decomposition of aromatic rings is expected above 500 °C [36]. The glass transition temperature (Tg) for soda lignin in our study was found to be at 109 °C. Additionally, the Tg value can be correlated with the molecular weight of lignin [51]. This value is in accordance with the values reported in the literature. The Tg for neat PLA is found to be at 71 °C in this study. The glass transition temperature of the PLA/Lignin biocomposites showed significant shift of the Tg from 71°C towards lower temperature, Tg of 59 °C for PLA/Lignin 20%. This can be explained by different molecular factors such as interchain hydrogen bonding, crosslinking density, rigid phenyl groups, and molecular mass [52]. 

### 3.4. Mechanical Properties

**Figure 3:**
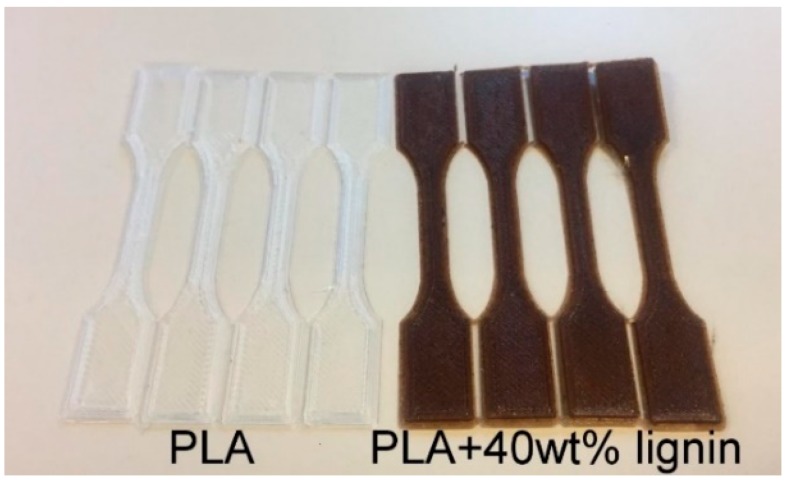
3D printed dogbones for mechanical testing.

**Figure 4:**
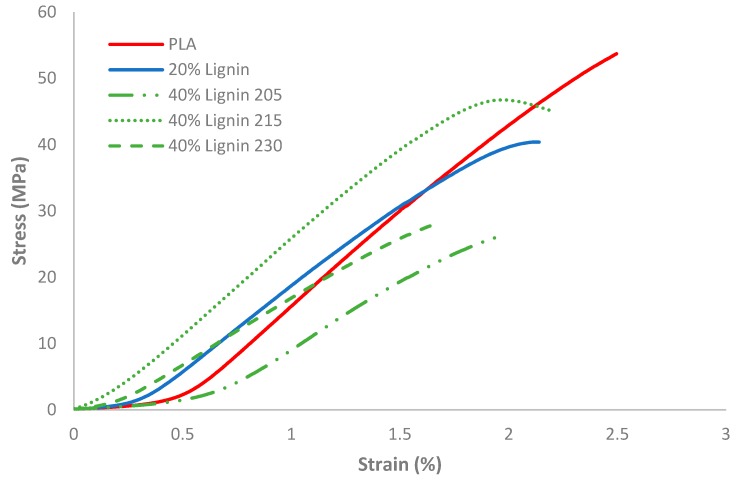
Stress−strain curves for the different biocomposites

The filaments (PLA, PLA/20%Lignin, and PLA/40%Lignin) were used to 3D print dogbones for further characterization (Figure 3). The stress−strain curves of the biocomposites with unmodified lignin showed that the mechanical strength decreased when the lignin content increased (Figure 4), which is in accordance with previous studies [21,22]. This is most probably due to a low fusing between the printing layers, especially in biocomposites with 40 wt % of lignin. However, when the printing temperature was increased to 215 °C, the biocomposite revealed a relative increase of the mechanical properties. This was presumptively due to an improved adhesion of the 3D printed layers. The strength−strain curves also show that lignin led to a more fragile biocomposite that resists less deformation before rupture. Consequently, the elastic modulus decreased by 25%–32%, compared to the neat PLA sample. The addition of low content of lignin in PLA biocomposites presented an improvement of the ductility. However, lignin content above 10% has caused a decrease in the plasticity of biocomposites, either for acetylated or unmodified lignin [53].

### 3.5. Scanning Electron Microscopy (SEM)

**Figure 5:**
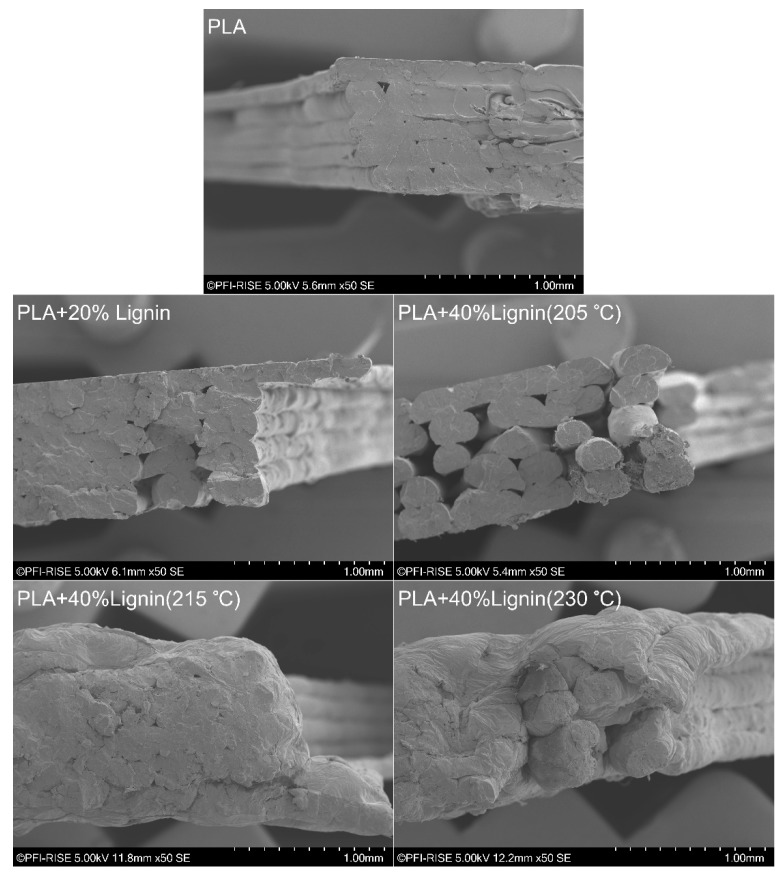
Scanning electron microscopy (SEM) analysis of the fracture surface of tensile tested dogbones.

The SEM images (Figure 5) of the fractured surface of the biocomposites provided an insight into the bonding interaction between lignin and PLA. The analysis of the PLA/40% Lignin sample revealed a 3D printed structure where the printed threads are clearly visible, thus confirming a poor inter-layer adhesion and a corresponding low mechanical performance (Table 1). In order to find a suitable printing temperature of the lignin-containing biocomposite and thus increase the inter-layer bonding, two additional temperatures were tested during the 3D printing process (215 °C and 230 °C). The results revealed that a suitable printing temperature for the biocomposite filaments developed in this study was 215 °C (Table 1 and Figure 4). The SEM pictures showed the improvement of the bonding of the printed layers, which favored the mechanical properties of the samples. However, increasing the printing temperature to 230 °C led to a decrease of the tensile strength and modulus. This was potentially caused by the degradation of the carbohydrates in the lignin fraction, which usually occurs over 220 °C, becoming volatile gases and presumptively creating microstructures within the polymeric matrix. Our results are in accordance with literature results of Thakur et al. [20] and Watkins et al. [50].

### 3.6. FT-IR Spectra and XRD Analysis

**Figure 6:**
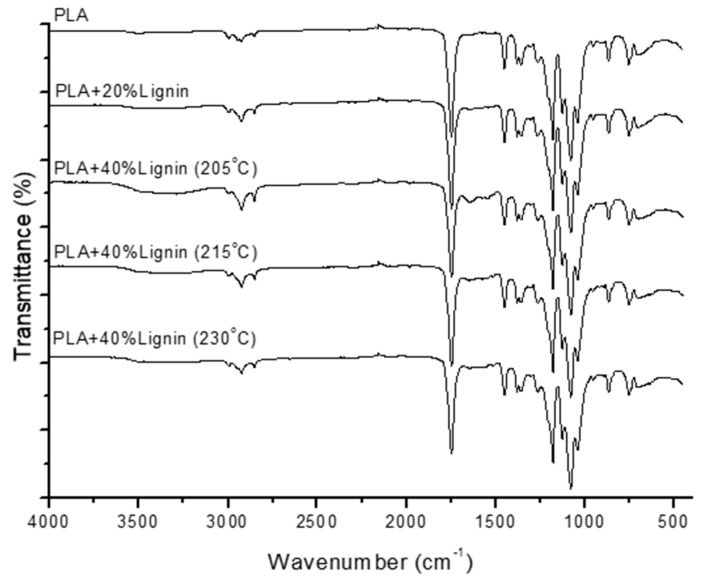
FT-IR spectra of PLA and PLA/lignin biocomposites.

FT-IR spectroscopy was applied to assess the functional groups of PLA/Lignin biocomposites at different temperatures. Generally, the curves of PLA/lignin showed similar bands with increased emissivity compared to neat PLA (Figure 6). Neat PLA showed peaks around 2995 cm^−1^ and 2930 cm^−1^, which are associated to the asymmetric and symmetric stretching vibration of CH_3_ group. The intense peak at 1749 cm^−1^ is attributed to the C=O stretching vibration [54]. The peak at 1450 cm^−1^ corresponds to CH_3_ anti-symmetric bending vibration. The peaks at 1385 cm^−1^, 1360 cm^−1^, 1316 cm^−1^, and 1300 cm^−1^ are associated to the deformation, symmetric, and bending mode of the CH group, respectively. The peaks at 1182 cm^−1^, 1084 cm^−1^, and 1038 cm^−1^ are attributed to C-O-C stretching vibrations [55]. This peak is obviously higher in the PLA/40%Lignin curve, which indicates that addition of lignin led to a higher content of hydroxy groups. In addition, the biocomposites containing lignin showed a small peak at 1510 cm^−1^ due to the C=C groups of the aromatic rings of lignin. 

**Figure 7:**
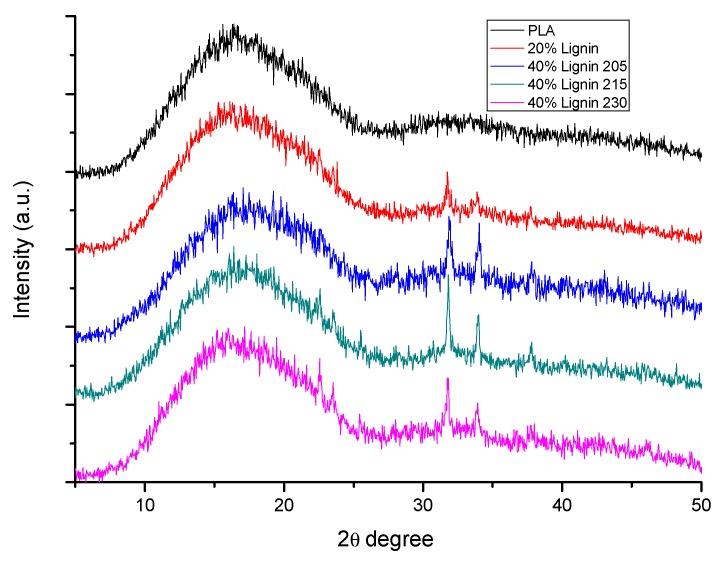
X-ray diffraction analysis (XRD) patterns of PLA and PLA/Lignin biocomposites.

X-ray analysis revealed a change in crystallinity as the lignin was included in the formulation (Figure 7). PLA exhibits a broad peak at 2θ degrees = 10°–25° associated with the semicrystalline nature of PLA. The appearance of peaks at 2θ = 32° and 34.5° in lignin-containing biocomposites indicated further crystallization of PLA, due to the action of lignin as nucleating agent [56].

### 3.7. Antioxidant Properties

**Figure 8:**
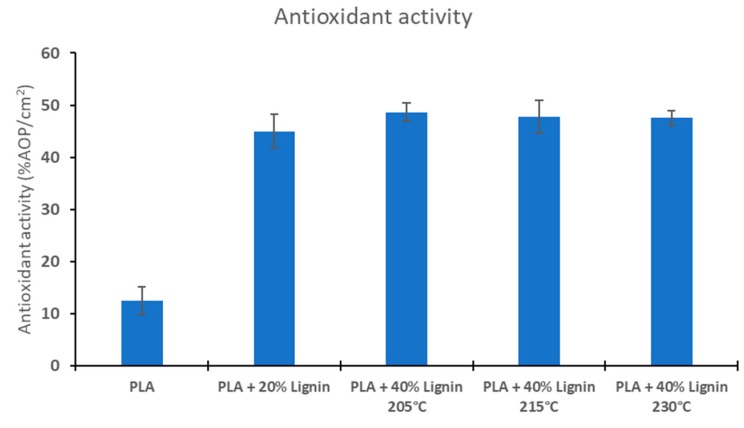
Antioxidant activity of PLA and biocomposites.

Antioxidant capacity of the biocomposites was measured and expressed as radical scavenging activity (RSA). PLA shows a low antioxidant (activity only associated with the surface interaction with the radical ABTS) (Figure 8). However, the lignin-containing biocomposites show a significantly higher radical scavenging activity (~50%) due to the antioxidant activity of lignin. Although, the biocomposites containing 40% lignin have a slightly higher RSA compared to the PLA/20% Lignin, the differences are not significant. The reaction of ABTS with the biocomposite material occurs mostly on the surface of the specimens, which may explain this behavior. The antioxidant activity of the lignin has been reported previously in several studies [45,57,58], including the blending of low amounts of kraft lignin (0.5 wt %–3 wt %) in PLA for potential biomedical applications [44]. Materials with high antioxidant activity are highly demanded for application in food packaging and biomedicine. A wide variety of antioxidant compounds are described in literature (resveratrol, curcumin, ascorbic acid, carotenoids, etc.), however, these compounds are generally expensive. For this reason, the use of lignin in biocomposites is proposed as a low-cost option to produce materials with high antioxidant capacity. 

Keep in mind that lignin is a natural antioxidant, and lignin has been proposed to stabilize a given material against photo- and thermo-oxidation [14,15,16,17,23]. The antioxidant property of soda lignin has been confirmed in this study where the printed materials containing lignin has a significant larger antioxidant capability compared to PLA (Figure 7). This suggests that the biocomposites developed in this study are also suitable for additional materials, e.g., food packaging applications. 

**Figure 9:**
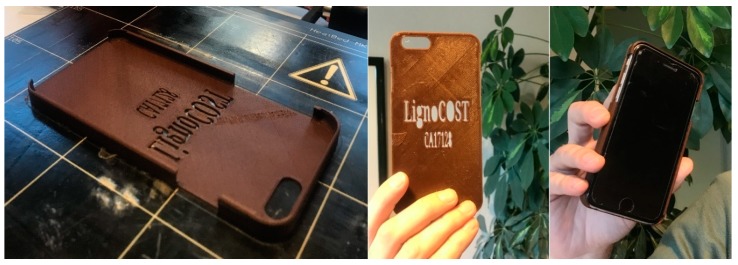
3D printing of a smartphone protective case with PLA/lignin biocomposite filament.

The suitability of the PLA/lignin biocomposite filament for 3D printing was also tested, by printing a smartphone protective case (Figure 9). The printing process revealed a good performance of the lignin-containing filament, and a functional protective case was effectively 3D printed. PLA/Lignin filaments are a plausible option for lignin utilization with potential in, e.g., rapid prototyping and consumer products [59]. It is worth to mention that the typical smell from some lignins was not detected during the extrusion of the filaments or during the printing process, which is an additional advantage of using soda lignin in PLA biocomposite materials. 

## 4. Conclusions

PLA/lignin biocomposites with different lignin loading were prepared and characterized in detail. Thermogravimetric analysis indicated that the thermal decomposition of lignin occurred over a wide temperature range. PLA/lignin biocomposites showed the highest antioxidant activity, due to the antioxidant activity of lignin. Biocomposites exhibited good extrudability and flowability with no observable agglomeration of the lignin. This suggests that lignin-containing biocomposites are plausible alternatives for 3D printing applications.

## Figures and Tables

**Table 1:** Mechanical properties of PLA and PLA/Lignin biocomposites.

3D Printed Sample	E_t_ (MPA)	σ_M_ (MPa)	Ꜫ_M_ %
PLA	2890 ± 14.14	58.45 ± 0.55	2.45 ± 0.10
PLA + 20%Lignin	2460 ± 155.56	39.35 ± 1.05	1.8 ± 0.10
PLA + 40%Lignin (205 °C)	1955 ± 19.92	32 ± 2.10	1.8 ± 0.20
PLA + 40%Lignin (215 °C)	2695 ± 148.49	45.65 ± 0.05	1.9 ± 0.08
PLA + 40%Lignin (230 °C)	1930 ± 183.85	29.25 ± 1.35	1.65 ± 0.10

E_t_ (tensile elastic modulus); σ_M_ (tensile strength); Ꜫ_M_ (elongation at tensile).
